# Comparison of sexual activity and life satisfaction in women with intended and unintended pregnancies

**Published:** 2016-01

**Authors:** Anahita Khodabakhshi Koolaee, Mahnaz Zamani, Leili Moslanejad, Safieh Jamali

**Affiliations:** 1 *Institute of Higher Education of Khatam, Tehran, Iran.*; 2 *Midwifery Clinic, Shahid Mostafa Khomaini Hospital, Shahhed Medical University, Tehran, Iran.*; 3 *Mental Health Department, Research Center for Social Determinant of Health, Jahrom University of Medical Sciences, Jahrom, Iran.*; 4 *Research Center for Social Determinants of Health, Jahrom University of Medical Sciences, Jahrom, Iran.*

**Keywords:** *Pregnancy unintended*, *Sexual activity*, *Personal satisfaction*

**Dear Editor**

Pregnancy and childbirth, as a part of the triple crises of life, such as puberty and marriage, are of considerable importance and could be matter of research. Like every other crisis, pregnancy is consisted of both physical and psychological changes. Besides, assisting in improvement of maternal health helps to understand these changes and their interactions, which create different clinical presentations in different people (1, 2).

There are rare studies regarding the satisfaction of life and sexual function in these individuals. Health care for mothers during pregnancy and prevention of fears, anxiety, and stress are essential issues. Moreover, following pregnancy, sanitation rules are the most important social requirements that include the following items: A) providing sanitary conditions during pregnancy with minimum physical and mental discomfort and maximum satisfaction and pleasure. B) delivery in the best possible position, having a healthy baby. C) providing the health of pregnant woman and guidelines for creating balance after childbirth (3).

In general, women with unwanted pregnancy have reported lower level of all dimensions of sexual function, like; sexual stimulation, orgasm, and satisfaction. Thus, it is confirmed that there is a significant difference in sexual function between women with intended pregnancy and those with unintended pregnancy in just two sub-scales that aforementioned above. There are several reasons that changes in sexual function occur during pregnancy, both in women with intended pregnancy, and those with unintended pregnancy (4).

There are certain critical stages in the life of every person that have a profound impact on the lives of individuals, including pregnancy that is one of the important periods of life. Because pregnancy for a woman looks like achieving sense of wholeness, perfection satisfies reproduction and the sense of being eternal. Although, it is an exceptional success in normal conditions, it makes the woman feeling happiness and satisfaction. So, she faces with many physical and behavioral changes (5).

The lower quality of the relationship between husband and wife in the family and lack of attachment to the family and generally disorders in marital relationship can bring resentment and hatred about pregnancy. On the other hand, sexual relationship in pregnancy period alters due to the numerous physical and psychological changes. In other words, the roles of men and women turn into those of the mothers and the fathers (6). 

Unintended pregnancy refers to pregnancies happen without desire or intention of couples or without preplanning. When faced with an unintended pregnancy, many of the women attempt to take irrational actions. They take care of themselves less than they used to and they are likely to take actions to terminate pregnancy (7).

Generally, unintended pregnancies occur for two reasons: non-use of contraceptives due to various personal and cultural reasons and the absence of fully effective contraceptive methods. According to one study, the women with unintended pregnancies suffer from lower psychological and physical recovery rates compared to women with intended pregnancies (8).

Due to the nature of their pregnancies, mothers with unintended pregnancy face with numerous risks during pregnancy such as: delay, decrease or failure in admitting the authorized centers to receive sanitary cares of pregnancy period (use of folic acid, iron, diagnosis and treatment of chronic diseases, diagnosis and treatment of eclampsia), also mothers with unintended pregnancy increasing physical and sexual violence against them (9).

The woman with intended pregnancy has long been waited for pregnancy and is happy and satisfied with her pregnancy. She prepares herself to be a mother and establishes an emotional relationship with her baby and gives special attention to the essential points that every pregnant woman should know and tries to accept her pregnancy and changes in family and social roles (10). 

Some studies have stated that women with unwanted pregnancy report higher physical and sexual abuse among their pregnancy and they decided to terminate their pregnancy. In addition, unintended pregnancy sometimes along with some strongly experience of Post-traumatic stress disorder (PTSD, symptoms of anxiety and depression. This associated symptoms impact the life satisfaction in unintended pregnancy (11).

The results demonstrate a clear relationship between a woman's experience of physical violence from her husband and her ability to achieve her fertility intentions. Also there is a relationship between unintended pregnancies and domestic violence.

Couples with unplanned pregnancies experience higher levels of sexual function problem before the birth of child. Couples with planned pregnancies have reported lower sexual problems during the pregnancy.

While some researchers, as mentioned before, indicated the lower sexual functions in all sub-scales and general scores in women with unintended pregnancies than the women with planned pregnancies, but our results in this study was inconsistent with those findings.

The women with unintended pregnancies reported lower sexual function in two scales than the women with intended pregnancy. In addition, there were differences in the life satisfaction in women with unintended pregnancies and women with intended pregnancy. Women with intended pregnancies experienced higher level of life satisfaction than women with unintended pregnancies. In general, the results indicated that for having a baby and enjoying the pregnancy period as a wonderful experienced that every mother has had, noticing the planned and continued the sexual function in couples is very important.

Based on the above studies and the fact that intended and unintended pregnancy can be effective as two basic components of life satisfaction in women’s lives, which means the sexual function and satisfaction, the researchers in this study tries to answer this question that what the difference is in sexual function and life satisfaction between intended and unintended pregnancy.
